# Polymeric micelles for the ocular delivery of triamcinolone acetonide: preparation and *in vivo* evaluation in a rabbit ocular inflammatory model

**DOI:** 10.1080/10717544.2020.1797241

**Published:** 2020-07-28

**Authors:** Mohamed A. Safwat, Heba F. Mansour, Amal K. Hussein, Soha Abdelwahab, Ghareb M. Soliman

**Affiliations:** a Department of Pharmaceutics, Faculty of Pharmacy, South Valley University, Qena, Egypt; b Department of Pharmaceutics, Faculty of Pharmacy, Minia University, Minia, Egypt; c Department of Histology and Cell Biology, Faculty of Medicine, Minia University, Minia, Egypt; d Department of Basic Medical Sciences, Deraya University, New Minia, Egypt; e Department of Pharmaceutics, Faculty of Pharmacy, Assiut University, Assiut, Egypt; f Department of Pharmaceutics, Faculty of Pharmacy, University of Tabuk, Tabuk, Saudi Arabia

**Keywords:** Triamcinolone acetonide, ocular inflammation, polymeric micelles, poly(caprolactone), ploy(lactic acid), chitosan

## Abstract

The aim of this study was to prepare triamcinolone acetonide (TA)-loaded poly(ethylene glycol)-*block*-poly(ε-caprolactone) (PEG-*b*-PCL) and poly(ethylene glycol)-*block*-poly(lactic acid) (PEG-*b*-PLA) micelles as a potential treatment of ocular inflammation. The micelles were evaluated for particle size, drug loading capacity and drug release kinetics. Selected micellar formulations were dispersed into chitosan hydrogel and their anti-inflammatory properties were tested in rabbits using a carrageenan-induced ocular inflammatory model. Particle size ranged from 59.44 ± 0.15 to 64.26 ± 0.55 nm for PEG-*b*-PCL and from 136.10 ± 1.57 to 176.80 ± 2.25 nm for PEG-*b*-PLA micelles, respectively. The drug loading capacity was in the range of 6–12% and 15–25% for PEG-*b*-PCL and PEG-*b*-PLA micelles, respectively and was dependent on the drug/polymer weight ratio. TA aqueous solubility was increased by 5- and 10-fold after loading into PEG-*b*-PCL and PEG-*b*-PLA micelles at a polymer concentration as low as 0.5 mg/mL, respectively. PEG-*b*-PLA micelles suspended in chitosan hydrogel were able to sustain the drug release where only 42.8 ± 1.6% drug was released in one week. TA/PEG-*b*-PLA micelles suspended in chitosan hydrogel had better anti-inflammatory effects compared with the plain drug hydrogel or the drug micellar solution. Complete disappearance of the corneal inflammatory changes was observed for the micellar hydrogel. These results confirm the potential of PEG-*b*-PLA micelles suspended in chitosan hydrogel to enhance the anti-inflammatory properties of triamcinolone acetonide.

## 1. Introduction

Uveitis is an inflammatory condition of the uvea, which comprises the iris, ciliary body and choroid (Guly & Forrester, 2010). Uveitis is one of the main causes of blindness and might be associated with systemic inflammation. It accounts for about 10% of blindness cases in people of working age in the Western societies (Suttorp-Schulten & Rothova, 1996). Uveitis can be classified into anterior, intermediate, posterior and panuveitis based on the anatomical location of ocular inflammation (Jabs et al., 2005). The etiology of uveitis varies according to the geographical location and includes infectious, noninfectious or ‘masquerade’ causes (Deschenes et al., 2008). The incidence of uveitis is around 20–50/100,000/year in Europe and USA with a prevalence around 100–150/100,000 population (Gritz & Wong, 2004). It most commonly affects the working age population with a mean age of incidence of 36 years (Çakar Özdal et al., 2014). The treatment options of uveitis depend on its etiology, the presence or absence of infection and the threat posed to sight (Guly & Forrester, 2010).

The standard therapy for noninfectious anterior uveitis is topical corticosteroids while posterior segment uveitis (intermediate, posterior, or panuveitis) is best treated by systemic corticosteroids (Krishna et al., 2017). Triamcinolone acetonide (TA) is one of the most commonly used corticosteroids in the management of ocular inflammation (Caceres-del-Carpio et al., 2016). Although eye drops are the most patient-friendly dosage form of TA, they suffer from many drawbacks such as short residence time, poor ocular bioavailability and frequent administration (Huang et al., 2018). TA in the form of subtenon, subconjunctival, or intravitreal injections have better therapeutic outcome but they also have several side effects such as acceleration of cataract formation, hemorrhage, retinal detachment, increased intraocular pressure and endophthalmitis (van Kooij et al., 2006). These drawbacks have motivated the development of innovative drug delivery systems that are able to achieve clinically effective ocular drug concentrations while limiting the side effects.

Throughout the past few decades, there has been much interest in the development of polymeric micelle formulations for ocular drug delivery due to their several intriguing features (Mandal et al., 2017). Polymeric micelles are formed by self-assembly of amphiphilic block copolymers in aqueous solutions. They have small size, in the nanometer range, which allows them to penetrate easily through ocular tissues and increase drug ocular bioavailability (Di Tommaso et al., 2012). Due to their unique core-shell architecture, hydrophobic drugs can be incorporated within the micelle core leading to increased aqueous drug solubility and enhanced hydrophobic drug transport across the hydrophilic stroma, which constitutes 85–90% of the cornea (Lee & Robinson, 1986). In addition, polymeric micelles made of mucoadhesive polymers such as chitosan or Pluronic^®^ F127 showed increased drug ocular residence time, which in turn resulted in enhanced drug efficacy (Lee & Robinson, 1986). Polymeric micelles based on polysaccharides such as hyaluronic acid and inulin have been used to incorporate various corticosteroids (dexamethasone, triamcinolone, triamcinolone acetonide). These micelles showed a promising ability to increase the *in vitro* and *ex vivo* drug permeability across corneal epithelial tissue compared with the drug suspensions (Bongiovi et al., 2017; Di Prima et al., 2017). Another interesting feature of polymeric micelles is that they can be designed to be biocompatible and/or biodegradable. Polyester-based polymers such as poly(ethylene glycol)-*block*-poly(ε-caprolactone) (PEG-*b*-PCL) and poly(ethylene glycol)-*b*-poly(lactic acid) (PEG-*b*-PLA) are examples of such biocompatible polymers that have been extensively used in the preparation of drug-loaded micelles (Ali et al., 2017; Binkhathlan et al., 2017; Grossen et al., 2017; Janagam et al., 2017). PCL and PLA blocks are well-known for their biocompatibility and biodegradability with slow degradation rate, which allows sustained ocular drug release (Kumar et al., 2001; Mahaling et al., 2018). To date, there are no published studies on using PEG-*b*-PCL or PEG-*b*-PLA micelles for the ocular delivery of TA.

Chitosan is a semisynthetic polysaccharide polymer consisting of repeating units of *N*-acetyl-d-glucosamine and d-glucosamine linked through β-(1-4) glycosidic bonds (Ahmed & Aljaeid, 2016). Chitosan hydrogels found several applications as ocular drug delivery systems due to chitosan mucoadhesive properties, which increase the ocular residence time and overcome rapid elimination of ophthalmic preparations. At physiological pH, chitosan carries positive charges, which further enhance its mucoadhesive properties through ionic interactions with ocular mucus and improve the ocular bioavailability of the incorporated drugs (Dubashynskaya et al., 2019). Furthermore, chitosan-coated polylactic-co-glycolic acid nanoparticles were able to augment the ocular anti-inflammatory properties of atorvastatin calcium (Arafa et al., 2020).

The aim of the present study was to prepare TA-loaded PEG-*b*-PCL and PEG-*b*-PLA polymeric micelles dispersed in chitosan hydrogel and test their abilities to enhance TA aqueous solubility, sustain its release and enhance its ocular anti-inflammatory activity. No other studies were carried out to enhance TA ocular anti-inflammatory properties using such polymeric micelles. Blank and drug-loaded micelles were prepared using the co-solvent evaporation method and evaluated using different techniques. The anti-inflammatory effect of TA-loaded micelles was evaluated, *in vivo* in a rabbit model of carrageenan-induced ocular inflammation.

## 2. Materials and methods

### 2.1. Materials

Triamcinolone acetonide was a gift from Amoun Pharmaceutical Company, Cairo, Egypt. Low molecular weight chitosan (M_W_ 50,000–190,000 Da, degree of deacetylation 75–85%) was purchased from Sigma-Aldrich Co. (St. Louis, MO). Dialysis membranes (Spectra/por, MWCO: 3.5–5 kDa, unless otherwise indicated) were purchased from Fisher Scientific (Rancho Dominguez, CA). The block copolymers poly (ethylene glycol)-*block-*poly(ε-caprolactone) (PEG2-*b*-PCL10) and poly (ethylene glycol)-*block-*poly (lactic acid) (PEG2-*b*-PLA1) were purchased from Polymer Source (Dorval, Canada). PEG2-*b*-PCL10 had a PEG molecular weight of 2 kDa (degree of polymerization ∼ 33) and PCL molecular weight of 10 kDa (degree of polymerization ∼ 88) while PEG2-*b*-PLA1 had a PEG molecular weight of 2 kDa and PLA molecular weight of 1 kDa (degree of polymerization ∼ 14). All other chemicals were reagent grade and used as received.

### 2.2. Methods

#### 2.2.1. Preparation of blank and drug-loaded polymeric micelles

Blank and TA-loaded polymeric micelles were prepared by the co-solvent evaporation method (Soliman et al., 2014a,b). Given weights of TA and each polymer (drug/polymer weight ratio of 0–50%) were solubilized in 2 mL of acetone. The obtained solution was then added in a dropwise manner (1 drop/10 s) to 4 mL of deionized water under magnetic stirring in a glass vial. The vials were kept open and the mixtures were magnetically stirred for 24 h to remove acetone and form the drug-loaded micelles. The unloaded drug was separated by filtration through a 0.45 μm PVDF filter. Samples of the drug-loaded micelle solution were used to determine the drug content by HPLC.

#### 2.2.2. HPLC assay of TA

TA concentration in the micellar solution was determined by a previously reported HPLC method (Sabzevari et al., 2013a). An Agilent Technologies HP 1100 chromatography system having a quaternary pump, a UV-visible diode array detector and a HP Vectra computer equipped with the HP-Chemstation software was used. The assay was conducted at 25 °C using a 250 × 4.6 mm column filled with 5 µm-reversed phase C18 Hypersil® BDS (Thermo, Bellefonte, PA). The flow rate was fixed at 1.0 mL/min of a 1:1 v/v mixture of acetonitrile-water. TA was detected at a UV wavelength of 241 nm and had a retention time ∼ 3.4 min. A calibration curve was constructed from a series of TA solutions in acetonitrile. The calibration curve was linear in the concentration range of 1 to 5 µg/mL (*r*^2^ ≥ 0.999).

#### 2.2.3. Determination of TA encapsulation efficiency and loading capacity

Aliquots of the micellar solution were diluted 10 times with acetonitrile to break the micelle structure and assayed by HPLC to determine their drug content. TA encapsulation efficiency and loading capacity were calculated from the following equations:

(1) $$\text{Loading capacity }(\text{weight \%})=\frac{\text{weight of TA in micelles}}{\text{weight of micelles tested}}\;\times 100\;$$

(2) $$\text{Encapsulation efficiency }(\text{weight \%})=\frac{\text{weight of TA in the micelles}}{\text{Initial weight of TA}}\;\times 100$$

#### 2.2.4. Determination of micelle size and polydispersity index

The micelle hydrodynamic diameter (*D*_h_) and polydispersity index (PDI) were determined using a Malvern ZetaSizer (Nano-ZS, Malvern Instruments, Worcestershire, UK). The instrument was equipped with a He-Ne laser operating at 633 nm and an avalanche photodiode detector. The samples were filtered through a 0.45 μm Millex Millipore PVDF membrane filter before the measurements. Measurements were done in triplicate at room temperature.

#### 2.2.5. Preparation of chitosan hydrogel containing TA or TA-loaded micelles

The required amount of chitosan was added gradually to aqueous acetic acid solution (1% v/v) and left overnight under magnetic stirring to swell and dissolve. The pH was adjusted to 7.4 using 0.5 M NaOH, which results in the formation of chitosan hydrogel (Deepthi & Jose, 2019). The chitosan concentration in the hydrogel was 1.8% w/v. Drug-loaded hydrogel was prepared by dispersing the required amount of TA or its corresponding PEG2-*b*-PLA1 micelles (drug/polymer ratio 40 wt.%) in the chitosan hydrogel. The drug concentration in all the hydrogel preparations was 0.0168% w/w.

#### 2.2.6. *In vitro* drug release studies

The *in vitro* release studies of TA from the micelles and micelles-loaded hydrogel were done adopting the membrane diffusion method (Soliman et al., 2017). A sample (1 mL for the micelles containing 0.168 mg TA or 1 gram for the hydrogel containing 0.168 mg TA) was accurately weighed and transferred to an open-end glass tube having a diameter of 3 cm. The lower end was closed with a standard semipermeable cellophane membrane (MWCO 12,000–14,000 Da) previously immersed in phosphate buffer pH 7.4 for 24 h using an elastic band. The glass tube was submerged in a 100 mL beaker containing 50 mL phosphate buffer pH 7.4 so that the surface of the cellophane membrane was placed just beneath the surface of the buffer solution. The beakers were placed in a water-bath shaker operating at 37 ± 0.5 °C and 50 rpm. At predefined time intervals, 1-mL samples were withdrawn and replaced with same volume of fresh buffer solution maintained at the same temperature. Drug content of the samples was determined spectrophotometrically at 247 nm.

#### 2.2.7. Assessment of ocular anti-inflammatory effect of TA micelles hydrogel

The ocular anti-inflammatory effect of TA-loaded PEG2-*b*-PLA1 micelles dispersed in chitosan hydrogel was investigated adopting a rabbit model of carrageenan-induced ocular inflammation. This study was approved by the Animal Ethics Committee of Minia University, Egypt and it adhered to the National Institutes of Health guide for the care and use of laboratory animals (NIH Publications No. 8023, revised 1978). Four groups, each of 4 healthy male New Zealand albino rabbits (weighing 2.5–3 kg) were used. Inflammation was induced by injecting 50 µL of 0.5% carrageenan solution in isotonic saline into the superotemporal quadrant using a 30-gauge needle that was inserted 2 mm posterior to the limbus. The first group was left untreated and served as a control. The second group was treated by plain TA hydrogel (50 µL twice daily). The third group was treated by TA micellar solution (50 µL twice daily) while the last group received TA micelles hydrogel (50 µL twice daily). TA concentration in all the preparations was 0.0168% w/v. The treatment was continued for 14 consecutive days, after which the rabbits were sacrificed. The eyeballs were removed, washed with phosphate buffer solution and fixed immediately with a 10% w/v formalin solution. Specimens were taken from the eyeballs, dehydrated with an alcohol gradient and placed in melted paraffin, which was solidified in block form. Sagittal sections of cornea (<5 µm) were cut, stained with hematoxylin and eosin (H and E), and then observed under microscope (×400) for pathological examination (El-Sayed et al., 2017).

#### 2.2.8. Immunohistochemistry studies

Sections of cornea were de-paraffinized by heating overnight at 60 °C and soaking in xylene followed by rehydration in descending grades of ethanol. Antigen retrieval was performed with citrate buffer (pH 6.0) at 97 °C for 20 min. Endogenous peroxidase activity was blocked by hydrogen peroxide in methanol at room temperature for 30 min. Nonspecific antigens were blocked by incubation in 0.3% bovine serum albumin in Tris-buffered saline/Tween 80 for 30 min. Slides were then incubated with anti-Ki-67 mouse monoclonal antibody (1:100 dilution) for 1 h at room temperature then over night at 4 °C. Sections were then washed 3 times, each for 5 min in buffer solution and incubated for further 30 min with biotinylated goat anti-rabbit secondary antibodies diluted 1:1000 followed by washing. The samples were incubated for 30 min with Vectastain ABC kits (Avidin, Biotinylated horse radish peroxidase complex) and washed for 10 min. The substrate, diaminobenzidine tetra hydrochloride (DAB) in distilled water was added for 5–10 min. The enzyme reaction was developed as described previously. The slides were lightly counterstained by Mayer’s hematoxylin. The positive control for Ki-67 was the tonsil, while the negative control was performed by omitting the step of primary antibody application (El-Domyati et al., 2017).

### 2.3. Statistical analysis

All the experiments were run in triplicate and the results were presented as mean ± SD. Analysis of the data was carried out using GraphPad Prism software version 5 (GraphPad Software Inc., La Jolla, CA). One-way analysis of variance (ANOVA) with Newman-Keuls post-hoc test was used to analyze the data. Differences with *p*-values less than .05 were considered significant.

## 3. Results and discussion

### 3.1. Preparation and characterization of TA-loaded micelles

**Figure 1.**
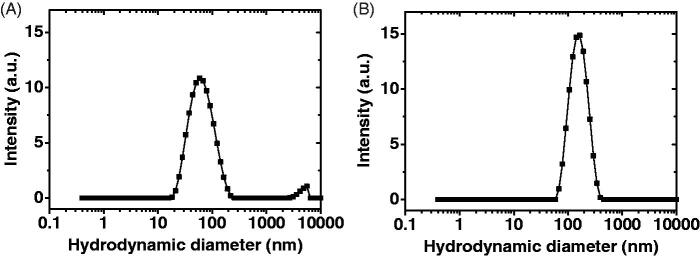
Distribution of the hydrodynamic diameter of (A) blank PEG2-*b*-PCL10 micelles, (B) blank PEG2-*b*-PLA1 micelles (solvent: deionized water; polymer concentration: 0.5 mg/mL).

TA-loaded micelles were prepared by the co-solvent evaporation method, which has proven useful in the preparation of drug-loaded micelles for several other drugs (Aliabadi et al., 2007; Soliman et al., 2014a,b). PEG2-*b*-PCL10 block copolymer was selected since our previous studies proved its excellent drug delivery properties in terms of small particle size, high drug loading capacity and sustained drug release (Soliman et al., 2014a). On the other hand, PLA is a hydrophobic polyester polymer that is approved for clinical use by the US-Food and Drug Administration (FDA) due to its biocompatibility and biodegradability (Wang et al., 2018). PEG-*b*-PLA has found applications as a delivery vehicle for several hydrophobic drugs due to its attractive features, such as improvement of hydrophobic drug solubility, biocompatibility and low uptake by the reticuloendothelial system (Xiao et al., 2010; Wang et al., 2018). Figure 1 shows that blank PEG2-*b*-PCL10 and PEG2-*b*-PLA1 micelles had unimodal size distribution with a hydrodynamic diameter of 59.44 ± 0.15 and 142.43 ± 2.21 nm, respectively (Table 1). The unimodal size distribution was further confirmed by a polydispersity index of 0.22 ± 0.03 for PEG2-*b*-PCL10 and 0.16 ± 0.02 for PEG2-*b*-PLA1 micelles, respectively (Table 1). It has been previously shown that a polydispersity index value smaller than 0.05 represents highly monodispersed populations whereas those larger than 0.7 represent heterogeneous size distribution (Danaei et al., 2018). Values of 0.2 or less are acceptable for polymeric nanoparticles and indicate homogenous population (Danaei et al., 2018).

**Table 1.** Hydrodynamic diameter (D*_h_*) and polydispersity index (PDI) of TA-loaded micelles.

TA/polymer ratio (wt.%)	PEG2-*b*-PCL10		PEG2-*b*-PLA1	
	D*_h_*	PDI	D*_h_*	PDI
0	59.44 ± 0.15	0.22 ± 0.03	142.43 ± 2.21	0.16 ± 0.02
10	59.36 ± 0.14	0.19 ± 0.02	136.10 ± 1.57	0.18 ± 0.02
20	61.14 ± 0.35	0.22 ± 0.00	153.30 ± 2.01	0.18 ± 0.04
30	59.27 ± 0.78	0.19 ± 0.02	176.80 ± 2.25	0.19 ± 0.02
40	64.26 ± 0.55	0.27 ± 0.02	146.90 ± 4.29	0.19 ± 0.03
50	60.22 ± 0.71	0.23 ± 0.01	148.23 ± 1.65	0.21 ± 0.06

### 3.2. Effect of drug/polymer ratio on the micelle size

Particle size is one of the most crucial attributes of polymeric micelles affecting not only their physical characteristics but also their *in vivo* performance as drug delivery vehicles (Yu & Qiu, 2016). Small sized nanoparticles could also increase drug permeation through biological membranes leading to enhanced bioavailability (Weng et al., 2017). In order to obtain TA-loaded polymeric micelles with optimum size and drug loading capacity, they were prepared at different TA/polymer weight ratios and the micelle size and drug loading properties were studies. Table 1 shows that the size of PEG2-*b*-PCL10 micelles was around 60 nm, regardless of the drug/polymer weight ratio or the level of drug loading. Likewise, the size of PEG2-*b*-PLA1 micelles was in the range of 136-176 nm and was not affected by drug loading or drug/polymer weight ratio. Some previous studies showed that drug encapsulation was accompanied by increase in micelle size due to micelle core expansion to accommodate the loaded drug (Liu et al., 2001; Soliman et al., 2014a). Others showed the opposite where drug loading resulted in decreasing the micelle size, which was attributed to decreasing the aggregation number of the polymer forming the micelles (Ahmad et al., 2014). It seems, therefore that the effect of drug loading on micelle size is dependent on various factors, such as the physicochemical properties of drug and polymer, the used drug/polymer ratio and the preparation method. The polydispersity index of both PEG2-*b*-PLA1 and PEG2-*b*-PCL10 micelles was around 0.2 confirming their monodispersity (Lu et al., 2011).

### 3.3. Effect of drug/polymer ratio and polymer type on the drug loading properties

TA loading capacity was dependent on the polymer type, as well as the drug/polymer weight ratio (Table 2). Thus, PEG2-*b*-PLA1 micelles had higher drug loading capacity compared with those of PEG2-*b*-PCL10 at all the tested drug/polymer ratios. The highest drug loading capacities were 25.18 ± 0.06 and 14.06 ± 0.10% for PEG2-*b*-PLA1 and PEG2-*b*-PCL10, respectively at a polymer concentration of 0.5 mg/mL. This represents TA aqueous solubility of 168.24 ± 0.5 and 81.08 ± 0.05 µg/mL, respectively. TA aqueous solubility is reported to be 17.5 µg/mL, which indicates that PEG2-*b*-PLA1 and PEG2-*b*-PCL10 micelles had around 10- and 5-fold increase in TA aqueous solubility at a polymer concentration as low as 0.5 mg/mL (Behl et al., 1976). The drug loading capacity observed in this study is higher than that observed for other drugs encapsulated into polymeric micelles, which typically falls in the range of 5–10% w/w (Kim et al., 2010; Shen et al., 2017). Other polymeric nanoparticle formulations, such as those of poly(_D,L_-lactide-co-glycolide) (PLGA) and poly β-amino ester showed low TA loading capacity in the range of ∼1–5% (Sabzevari et al., 2013a,b). High drug loading capacity observed in this study is advantageous since it limits the needed amounts of carrier polymers, which in turn limits the unnecessary burden of degrading, metabolizing and excreting the polymers from the body (Shen et al., 2017).

**Table 2.** Drug loading properties of PEG2-*b*-PCL10 and PEG2-*b*-PLA1 micelles.

TA/polymer ratio (wt.%)	PEG2-*b*-PCL10		PEG2-*b*-PLA1	
	LC^a^	EE^b^	LC^a^	EE^b^
10	6.78 ± 0.09	72.70 ± 1.01	9.97 ± 0.50	99.75 ± 0.48
20	14.06 ± 0.10	81.82 ± 0.05	15.03 ± 0.09	88.42 ± 0.63
30	12.65 ± 0.06	48.28 ± 0.27	17.07 ± 0.03	68.62 ± 0.13
40	11.45 ± 0.11	32.34 ± 0.34	25.18 ± 0.06	84.12 ± 0.25
50	11.19 ± 0.04	25.21 ± 0.11	17.82 ± 0.10	43.38 ± 0.28

^a^Percent drug loading capacity determined from Equation (1).

^b^Percent drug encapsulation efficiency determined from Equation (2).

The drug encapsulation efficiency was also higher for PEG2-*b*-PLA1 micelles compared with those of PEG2-*b*-PCL10 (Table 2). For both micellar systems, the drug encapsulation efficiency was highest at lower drug/polymer ratio and it started to decrease with the increase in the ratio. It is reported that these micelles have a certain drug encapsulation efficiency above which excess drug is precipitated without being loaded into the micelles. Therefore, the increase in the drug/polymer ratio increased the free (unloaded drug) leading to lower encapsulation efficiency according to Equation (2) (Soliman et al., 2014a). The reason for higher TA loading and encapsulation efficiencies for PEG2-*b*-PLA1 micelles compared with those of PEG2-*b*-PCL10 is not clear and requires further investigation. It was previously shown that drug loading properties of block copolymer micelles were dependent on several factors, such as physicochemical properties of the drug and copolymer, presence of specific interactions between the drug and the core-forming block and method of micelle preparation (Liu et al., 2006). The compatability between a given drug and polymer mixture is another important factor that influences the drug loading capacity (Liu et al., 2004; Sharma et al., 2012). This compatibility is based on the Hansen partial solubility parameter where similar parameters for a given drug and polymer were previously shown to result in high drug loading capacity (Liu et al., 2004; Sharma et al., 2012). In light of the above, it could be assumed that higher loading capacity observed for PEG2-*b*-PLA1 micelles might be due to better compatibility between TA and PLA polymer segments. Based on these results, PEG2-*b*-PLA1 micelles (drug/polymer weight ratio of 40%) were selected for further drug release and *in vivo* studies due to their higher drug loading capacity. It is noteworthy that these micelles have a drug loading capacity of 25.18 ± 0.06%, which is much higher than the highest value obtained for PEG2-*b*-PCL10 micelles (14.06 ± 0.10%, Table 2). The high drug loading capacity is of prime importance for clinical applications to maximize the drug/excipients ratio and minimize the hazards associated with the use of unnecessary chemicals. Although data in Table 1 shows that the size of PEG2-*b*-PLA1 micelles (146.90 ± 4.29 nm) is larger than that of PEG2-*b*-PCL10 micelles (64.26 ± 0.55 nm), the former is still in the nanometer range and has the potential to enhance drug efficacy (Hanafy et al., 2019).

### 3.4. *In vitro* drug release studies

**Figure 2.**
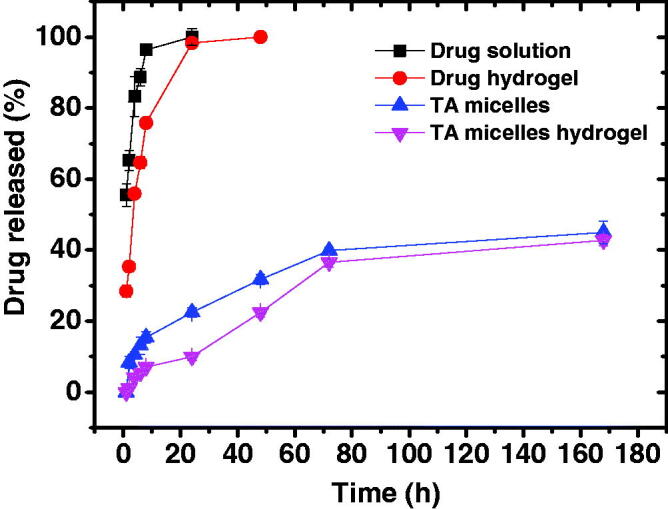
Cumulative percent of TA released from different formulations in phosphate buffer pH 7.4 at 37 °C.

Figure 2 shows the cumulative percent drug released from TA aqueous suspension and PEG2-*b*-PLA1 micelles in comparison with the same preparations incorporated into chitosan hydrogel. About 95% of the drug was released from its aqueous suspension in 8 h. Drug release from the aqueous suspension includes two steps; drug dissolution and drug diffusion through the dialysis membrane. This experiment was carried out under sink conditions which explains the relatively rapid dissolution of this hydrophobic drug (Behl et al., 1976). In contrast, the drug suspended in chitosan hydrogel showed significantly slower drug release rate up to 8 h (*p* < .05). The next sample taken at 24 h showed that the cumulative amount of drug released was almost the same for both the drug suspension and hydrogel, probably because the hydrogel had more time (16 h) for drug dissolution and release. TA release from the hydrogel was slower since drug release involved three steps; TA dissolution, drug diffusion through the hydrogel and finally drug diffusion through the dialysis membrane (Mourtas et al., 2007; El-Sayed et al., 2017). Incorporation of the drug into the micelles resulted in further significant reduction in the drug release rate. Thus, after 24 h the cumulative amount of TA released from PEG2-*b*-PLA1 micelles was significantly smaller than that released from the drug suspension or the drug dispersed in chitosan hydrogel (*p* < .05). After one week, only 45 ± 3.2% of the drug loaded into the micelles was released. TA is a hydrophobic compound with a Log p value of 2.5 (Thakur et al., 2011). This hydrophobicity facilitates hydrophobic interactions with the PLA segment in the micelle core, which limit the drug release to the surrounding aqueous media. Previous studies showed that TA entrapment into (polylactide – polycaprolactone – polyethylene glycol – polycaprolactone – polylactide) pentablock copolymers significantly slowed down its release rate due to hydrophobic interactions between the drug and the hydrophobic segments of the copolymer (Tamboli et al., 2013). Incorporation of TA micelles into chitosan hydrogel resulted in further reduction in the drug release rate. The cumulative amount of TA released from the micelle-loaded hydrogel was significantly smaller than that released from the micelles for up to 72 h (*p* < .05), after which the difference was non-significant, probably due to gel dissolution. Thus, after one week the cumulative amount of drug released from TA micelle-loaded hydrogel was 42.8 ± 1.6% compared with 45 ± 3.2% for the micelles (*p* > .05). Similar results have been previously reported for flurbiprofen niosomes dispersed into Carbopol 934 gel and was attributed to the presence of additional diffusion barrier for the drug to be released from the hydrogel (El-Sayed et al., 2017).

### 3.5. *In vivo* studies

#### 3.5.1. Confirmation of inflammatory changes in the rabbits' cornea

**Figure 3.**
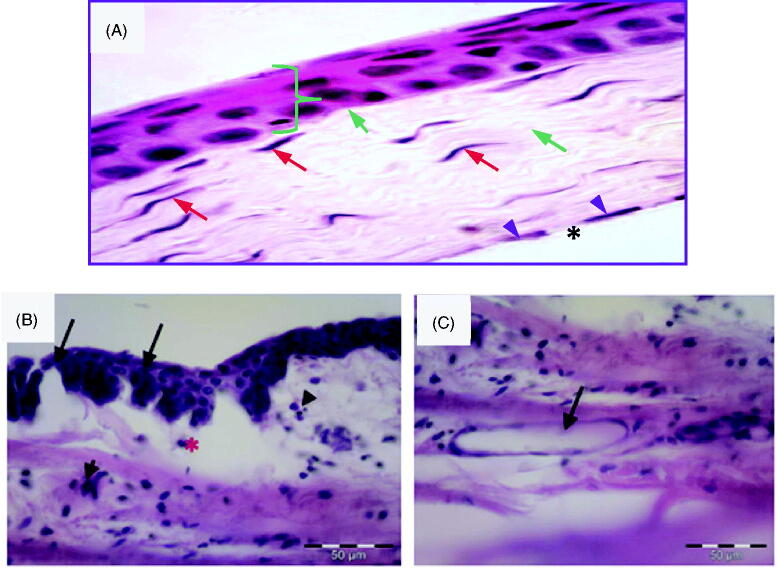
**(**A) Photomicrograph of control normal corneal tissue showing stratified squamous non- keratinized epithelium resting on basal lamina (}). Bowman’s layer appears as acellular, condensed region of the apical stroma (blue arrow). Stroma contains keratocytes (red arrow) within the acidophilic parallel regular lamella of stromal collagen (green arrow). Endothelial cell nuclei are seen in a single layer (arrow head) beneath the Descemet’s membrane (*). (H &E × 400). (B) Photomicrographs of the corneal tissue of adult male albino rabbits 2 h after carrageenan injection showing disfigured epithelium (arrow) with inflammatory cell infiltration (arrowhead). Notice edema and disorganization of the stroma (*). (C) Stroma of the cornea 2 h after carrageenan injection showing inflammatory cell infiltration and endovascular formation (arrow). H&E scale bar 50 µm.

Carrageenan solution in isotonic saline (50 µL, 0.5%) was injected into the superotemporal quadrant of the cornea. In order to confirm the inflammatory changes in the cornea, histological samples were obtained from rabbits sacrificed 2 h after injection. Corneal tissue samples obtained from rabbits that did not receive any injections were used as a control. Control normal corneal tissue (Figure 3(A)) shows different normal structures: epithelium, Bowman’s membrane, stroma, Descemet’s membrane and endothelium. The epithelium is stratified squamous non-keratinized tissue having five layers: the basal layer (a single layer of columnar cells), two layers of wing cells (shaped like a wing), 3–4 superficial layers of squamous cells with flattened nuclei and Bowman’s layer (non-cellular clear membrane). Its anterior surface toward the corneal epithelium is smooth, while the posterior one is irregular and interweaved with fibrils of anterior stroma. The stroma is formed of lamellar collagen fibers regularly arranged in parallel way. Long slender keratocytes (corneal fibroblasts) are present between collagen fibers and they are flat cells with thin cytoplasm and elongated nuclei. Descemet’s membrane appears as homogenous acidophilic layer. Corneal endothelium is formed of single layer of flat cells with flat nuclei. Figure 3(B,C) show photomicrographs of the corneal tissue of adult male albino rabbits 2 h after injection of carrageenan solution in isotonic saline (50 µL, 0.5%) into the superotemporal quadrant. The corneal tissue stained with hematoxylin and eosin showed manifestations of acute inflammation in the form of disfigured epithelium, pyknotic nuclei, loss of Bowman’s membrane and epithelial loss. The stroma showed inflammatory cell infiltration, edema, collagenolysis, defect formation and neovascularization (Figure 3(B,C)). These results agree with previous reports, which found that different degrees of corneal edema, opacity and infiltration of inflammatory cells were observed in corneal tissues between 3 and 24 h after lipopolysaccharide treatment (Chen et al., 2015).

#### 3.5.2. Anti-inflammatory studies

**Figure 4.**
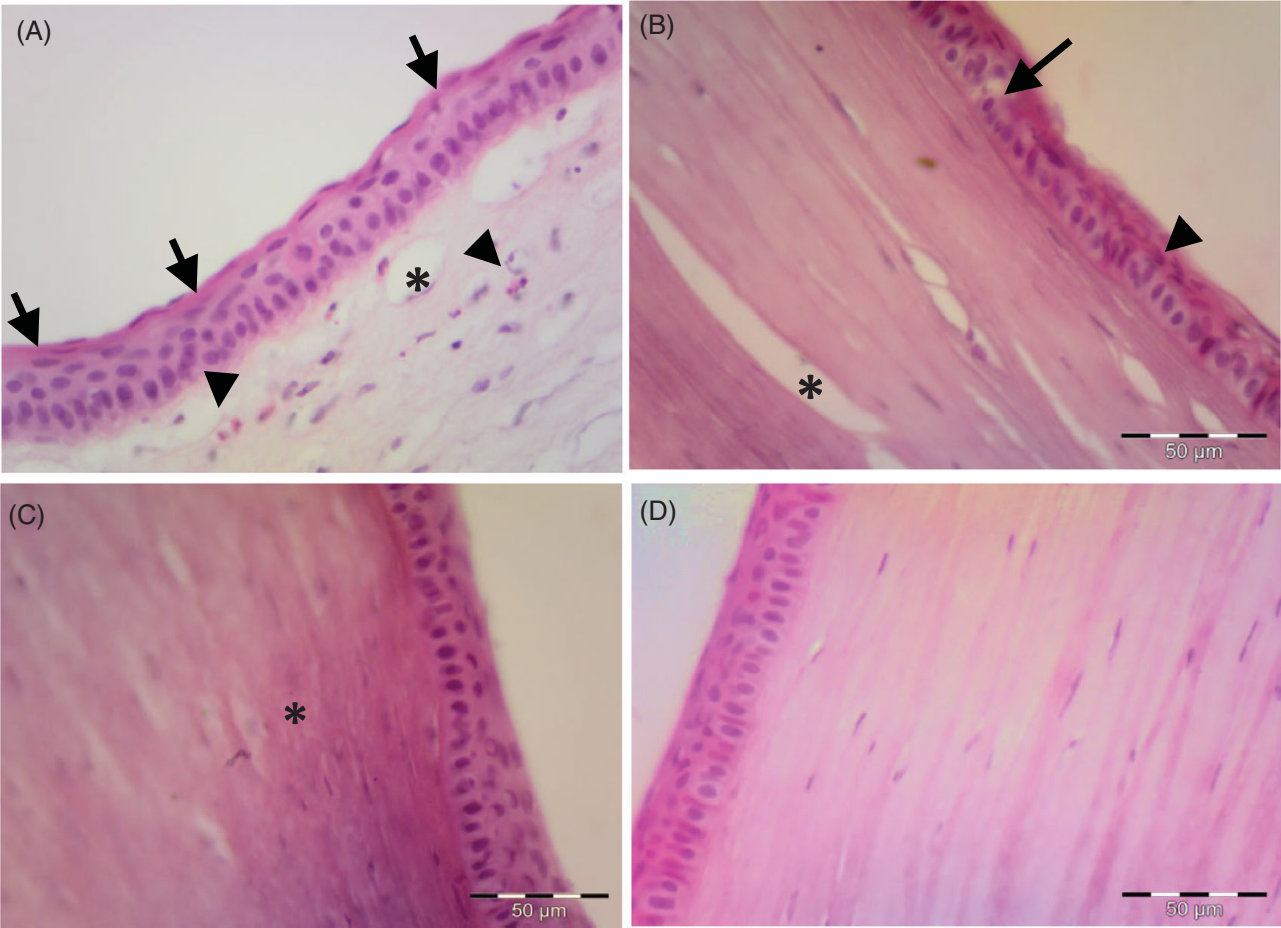
Photomicrograph of the corneal tissue of adult male albino rabbits 14 days after carrageenan injection. (A) Group I, non-treated cornea showing increase in epithelial thickness and appearance of horny superficial layer on the corneal surface (arrow), inflammatory cells could be observed in the stroma (arrowhead), as well as newly formed blood vessels (*). (B) Group II (plain drug hydrogel) showing vacuolated epithelial cells (arrow), eosinophilic cytoplasm of others (arrowhead) and stroma showing improved organization of collagen (*). (C) Group III (micellar solution) showing nearly normal epithelial pattern. Stroma showing homogenization of collagen (*). (D) Group IV (TA micelles/chitosan hydrogel) showing nearly normal appearance of the cornea with normal epithelial arrangement and normal stromal pattern. H&E scale bar 50 µm.

Figure 4 shows photomicrographs of the corneal tissue of adult male albino rabbits 14 days after carrageenan injection. The cornea of non-treated group (group I, Figure 4(A)) showed increased epithelial thickness and appearance of horny superficial layer on the corneal surface. Inflammatory cell infiltration, collagen disorganization and neovascularization were observed in the stroma. The group treated with plain drug hydrogel (group II, Figure 4(B)) showed vacuolation of some epithelial cells while others showed eosinophilic cytoplasm and apoptotic figures. Inflammatory cell infiltration was less apparent compared with the untreated control, but epithelial dystrophy and collagen disorganization were still present. The corneal tissue in the group treated with TA incorporated into PEG2-*b*-PLA1 micellar solution (group III, Figure 4(C)) showed improved histological architecture of the cornea except some areas of collagen dystrophies observed in the stroma. Nearly normal epithelial pattern was observed. Homogenized collagen fibers were observed in the stroma (Couch & Bakri, 2009). This enhanced anti-inflammatory effect observed for TA micelles compared with the free drug hydrogel might be attributed to better drug penetration achieved by the micelle small size. Similar results were previously reported by Sabzevari et al. where TA incorporated into PLGA nanoparticles and lipid nanocapsules had better anti-inflammatory effect compared with the drug suspension (Sabzevari et al., 2013b; Guo et al., 2019; Formica et al., 2020). Group IV treated with TA-loaded PEG2-*b*-PLA1 micelles incorporated into chitosan hydrogel showed normal epithelial thickness of the corneal tissue and normal epithelial pattern. Stroma showed well-organized collagen fiber with normal keratocytes (Figure 4(D)). Complete disappearance of inflammatory changes was observed and the corneal histological features had normal structure.

#### 3.5.3. Immunohistochemistry studies

**Figure 5.**
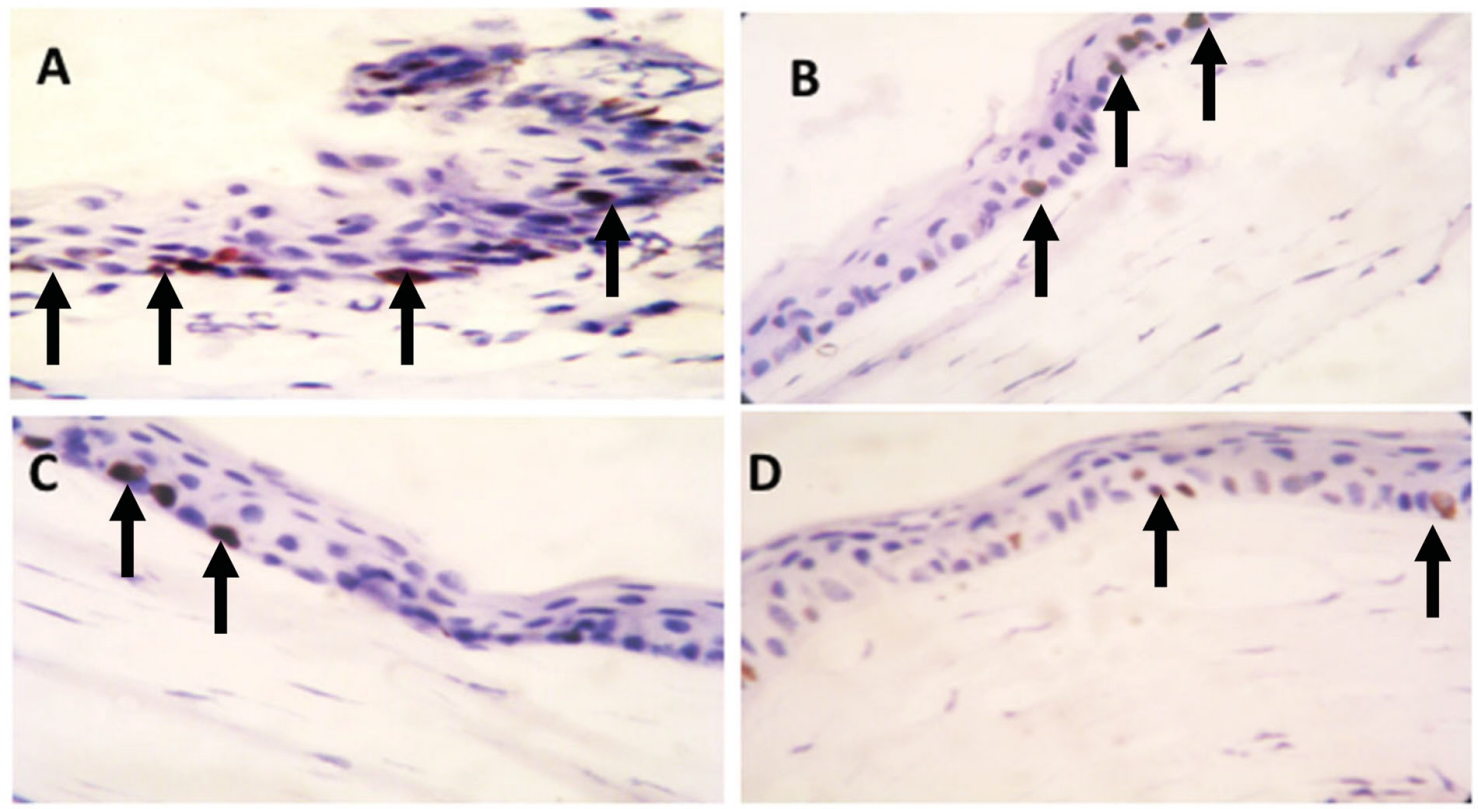
Ki-67 immunostaining of the corneal tissue of adult male albino rabbits 14 days after carrageenan injection. (A) Group I (non-treated group) showing many cells expressing ki-67 (arrow). (B) Group II (plain drug hydrogel) showing some positive cells for ki-67 (arrow). (C) Group III (micellar solution) showing few positive cells expressing ki-67 (arrow). (D) Group IV (TA micelles/chitosan hydrogel) showing that the expression of ki-67 is rare and very low (arrow) (Counter stain hematoxylin ×40).

To investigate cell proliferation in the peripheral cornea, immunohistochemistry using ki-67 was done (Martin et al., 2013). Ki-67 is a widely used protein as a marker for cell proliferation (Cuylen et al., 2016). The positive immuno-reactivity appears as brown stained nuclei of immuno-positive cells. Fourteen days after injury, the immuno-positive nuclei were numerous in non-treated peripheral cornea (group I, Figure 5(A)). This might explain the increase in the thickness of epithelium and appearance of horny layer. The immuno-positive cells were less numerous in case of the group treated with the plain drug hydrogel (group II, Figure 5(B)). Furthermore, the group treated with TA-loaded PEG2-*b*-PLA1 micellar solution had few immuno-positive nuclei (group III, Figure 5(C)). Interestingly, the number of immuno-positive nuclei in the peripheral cornea of the group treated with the TA-loaded PEG2-*b*-PLA1 micelles suspended in chitosan hydrogel (group IV) was very scarce (Figure 5(D)). These results are in agreement with previous reports which showed that mucoadhesive nanoparticles of TA had better anti-inflammatory effect in the rabbit eyes compared with the drug suspension (Sabzevari et al., 2013a). The enhanced anti-inflammatory efficacy of TA micelles suspended in chitosan hydrogel compared to all other formulations could be attributed to several factors. First, chitosan hydrogel is well-known for its mucoadhesive properties, which result in prolonged ocular residence time and consequent enhanced ocular drug efficacy (Dubashynskaya et al., 2019). Second, the micelle small nanometric size might lead to better drug penetration through the corneal tissue and better drug bioavailability. Chitosan has its mucoadhesive properties through electrostatic interactions with sialic acid residues of mucin, as well as non-covalent interactions with mucin (Irimia et al., 2018). Due to these interesting properties, different ocular preparations of chitosan have shown excellent potential in enhancing the efficacy and bioavailability of several other drugs (Başaran & Yazan, 2012; Mohammed et al., 2017; Silva et al., 2017; Khan et al., 2018).

## 4. Conclusion

TA was successfully loaded into polyester-based micelles of PEG2-*b*-PLA1 and PEG2-*b*-PCL10 block copolymers. Both micellar systems showed high drug loading and drug encapsulation efficiencies that were dependent on the drug/polymer weight ratios and were higher for PEG2-*b*-PLA1 micelles. The micelles with the highest drug loading capacity were suspended in chitosan hydrogel in order to increase the drug ocular residence time and sustain its release. The micelles suspended in the hydrogel were able to significantly slow down the drug release rate where only ∼42% of the drug was released in one week compared with ∼95% for the drug suspension after 8 h. When tested *in vivo* in a carrageenan-induced ocular inflammatory model in rabbits, PEG2-*b*-PLA1 micelles suspended in chitosan hydrogel were able to abate ocular inflammation and normal corneal histology was recovered. Results from this study confirm that PEG2-*b*-PLA1 micelles dispersed in chitosan hydrogel could serve as an efficient ocular delivery system to enhance TA anti-inflammatory effects and prolong its release.
